# 

**Published:** 1823-02

**Authors:** 


### MONTHLY CATALOGUE OF MEDICAL BOOKS.

*** *It having been hinted to us that gentlemen, sending copies of their Works, prefer having their titles given at length, it is our intention, hereafter, to comply with this suggestion: but it is to be observed, that no books can be entered on this list except those sent to us for the purpose;—in the lists hitherto transmitted, we find that the names of works have frequently been given as published, which have not appeared for weeks, or even months after.*

Observatio de Affectibus Morbosis Virginis Haviniensis cui Plurimæ acus e variis Corporis partibus excisæ et extractæ sunt. Auctore J. D. Herholdt, Medicinæ Doctore et Professore, protomedico Nosocomii Regii Fredericiani, Auctore Medicinæ Militaris Nauticæ, Equite ordinis Danebrogici, Membro plurium Societatum Doctarum.—1822. pp. 44.

An Essay on the Absorbent Vessels, showing that their Action is not liable to be influenced by the artificial Agents commonly applied. By Henry Searle, Surgeon.—1823. pp. 26.

An Essay on the Medicinal Efficacy and Employment of the Bath Waters, illustrated by Remarks on the Physiology and Pathology of the Animal Frame, with Reference to the Treatment of Gout, Rheumatism, Palsy, and Eruptive Diseases. By Edward Barlow, M.D. Graduate of the University of Edinburgh, Member of the Royal College of Surgeons of Ireland, one of the Physicians of the Bath Hospital, and of the Bath City Infirmary and Dispensary, and Physician of the Charitable Society for the Relief of Lying-in Women.—Bath, 1822. pp. 200.

An Appendix to the Midland Flora; comprising also Corrections and Additions referring to the two former Volumes, and occasional Observations tending to elucidate the Study of the British Fungi; concluding with a general Index to the whole Work, and a general Index of Synonyms. In two Parts. By THOMAS PURTON, F.L.S. Member of the Royal College of Surgeons. Embellished with thirty coloured Engravings. Vol. III. Part I. and II.—1821. pp. 575.

---

### METEOROLOGICAL JOURNAL.

